# A panel of 13-miRNA signature as a potential biomarker for predicting survival in pancreatic cancer

**DOI:** 10.18632/oncotarget.11903

**Published:** 2016-09-08

**Authors:** Xin Zhou, Zebo Huang, Lei Xu, Mingxia Zhu, Lan Zhang, Huo Zhang, Xiaping Wang, Hai Li, Wei Zhu, Yongqian Shu, Ping Liu

**Affiliations:** ^1^ Department of Oncology, First Affiliated Hospital of Nanjing Medical University, Nanjing 210029, China; ^2^ Department of Thoracic Surgery, The Affiliated Jiangning Hospital of Nanjing Medical University, Nanjing 210029, China; ^3^ Department of Pathology, Sir Run Run Hospital Affiliated With Nanjing Medical University, Nanjing 211166, China; ^4^ Department of Pathology, First Affiliated Hospital of Nanjing Medical University, Nanjing 210029, PR China; ^5^ Cancer Center of Nanjing Medical University, Nanjing 210029, China

**Keywords:** pancreatic cancer, miRNA, signature, biomarker, prognosis

## Abstract

Some reports have evaluated the prognostic relevance of microRNAs (miRNAs) in patients with pancreatic cancer (PC). However, most studies focused on limited miRNAs with small number of patients. The aim of the study is to identify a panel of miRNA signature that could predict prognosis in PC with the data from The Cancer Genome Atlas (TCGA). A total of 167 PC patients with the corresponding clinical data were enrolled in our study. The miRNAs significantly associated with overall survival (OS) in PC patients were identified with Cox proportional regression model. A risk score formula was developed to evaluate the prognostic value of the miRNA signature in PC. Thirteen miRNAs were identified to be significantly related with OS in PC patients. Patients with high risk score suffered poor overall survival compared with patients who had low risk score. The multivariate Cox regression analyses showed that the miRNA signature could act as an independent prognostic indicator. In addition, the signature might serve as a predicator for treatment outcome. Our study identified a miRNA signature including 13 miRNAs which could serve as an independent marker in prognosis of PC.

## INTRODUCTION

Pancreatic cancer (PC) is one of the most fatal malignancies with increasing incidence and high mortality all around the world. Less than 10% PC patients are diagnosed at an early stage, and most patients do not have opportunity of surgical resection due to relatively late stage [1]. Moreover, more than 50% of cases who receive surgery still suffer recurrence within 12 months because of highly aggressive nature of PC [2]. Despite recent advances and efforts in the treatment, the 5-year overall survival (OS) rate of PC is lower than 5% [3]. Predicting prognosis of PC patients might be helpful to choose more suitable treatment strategies thus leading to improved clinical outcomes.

Currently, traditional factors such as tumor grade and TNM stage are used to guide treatment and predict survival in the clinical. Serum carbohydrate antigen 19–9 (CA 19–9) remains to be the only biomarker to monitor disease progression during PC treatment [4]. However, there often exists inconsistence between these predictors and survival [5, 6]. The limitation of CA 19–9 such as poor specificity, negative results in Lewis negative phenotype and false positive elevation in the presence of obstruction jaundice also restricts its role in the clinical [7]. Thus, finding prognostic biomarkers that might improve clinical outcome through patient classification and increase further understanding of the mechanisms of PC is highly desirable.

MicroRNAs (miRNAs) are a family of short (typically 18–25 nucleotides), single-stranded and highly conserved non-coding RNAs which could suppress gene expression at post-transcriptional level through base pairing with the 3′-untranslated region of target mRNAs, resulting in either mRNA degradation or translational repression [8, 9]. Increasing evidence demonstrated that miRNAs could play important roles in various biological processes, such as cellular development, metabolism, differentiation, proliferation and angiogenesis [10–13]. MiRNAs could also participate in the development and progression of cancer by acting as tumor suppressors or onco-miRNAs. Recently, many studies explored the value of miRNAs as biomarkers for diagnosis, prognosis or monitoring curative effect in various cancers [14–18]. The prognostic value of miRNAs in PC has been identified in some previous studies [19]. However, most studies focused on miR-21, with few reports regarding the effect of other miRNAs on outcomes in PC patients. In addition, the number of patients enrolled in these studies is generally small. Here, we conducted this study using the dataset retrieved from The Cancer Genome Atlas (TCGA, http://cancergenome.nih.gov/) to identify a panel of miRNA signature which could predict prognosis in PC.

## RESULTS

### Patient characteristics

A total of 167 PC patients were included in our study. Clinical features were summarized in Table 1. The mean ± standard deviation (SDV) age for all patients was 64.6 ± 11. During the follow-up (mean ± SDV: 12.8 ± 15.5 months), 57 of 167 (34.1%) patients died. Information on treatment outcome of first course was available in 92 patients, of which 39 (42.4%) achieved complete response (CR), 8 (8.7%) partial response (PR), 7 (7.6%) stable disease (SD) and 38 (41.3%) progressive disease (PD).

**Table 1:** Characteristics of 167 PC patients enrolled in the study

Characteristics	Number
All	167
Gender, male/female	92/75
Age, < 65/≥ 65	78/89
Location, head/body/tail/others	132/11/13/11
Size (mm), < 35w≥ 35	79/91
Grade, G1/G2/G3/G3/GX	30/87/47/2/1
Residual tumor, R0/R1/R2/RX/NA	103/51/2/4/7
TNM stage, IA/IB/IIA/IIB/III/IV/NA	7/13/25/114/4/3/1
Smoker, no/yes/NA	60/76/31
Drinker, no/yes/NA	94/29/44
Diabetes, no/yes/NA	101/36/30
Adjuvant radiotherapy, no/yes/NA	94/29/44
MTT, no/yes/NA	44/81/42

### Identification of miRNAs associated with OS

According to the exclusion criterion, 339 of 1046 miRNAs were selected for further analysis. And a total of 13 miRNAs (10 protective miRNAs: miR-103-2, miR-125a, miR-126, miR-328, miR-340, miR-361, miR-374b, miR-454, miR-627 and miR-664 and 3 risky miRNAs: miR-193b, miR-21 and miR-584; Supplementary Table S1 online) were identified to be correlated with OS in PC patients. The association of the miRNAs with clinical features was assessed (Table 2). MiR-361 was found to be related with gender, age and smoking in PC patients. The oncogenic miR-21 was significantly associated with tumor size and stage which was also positively correlated with miR-193b. Interestingly, five miRNAs (miR-103-2, miR-126, miR-340, miR-374b and miR-627) were related with alcohol consumption. No other association was found between the miRNAs and clinical factors.

**Table 2:** The association of the 13 miRNAs and the miRNA signature risk score with clinical features in PC patients (presented as *P* value)

ID	Gender (female VS. male)	Age (< 65 VS. ≥ 65)	Grade (G1 + G2 VS. G3 + G4)	Tumor size (< 35 mm VS. ≥ 35 mm)	Stage (I vs. II VS. III + IV)	Smoking (smoker VS. non-smoker)	Drinking (drinker VS. non-drinker)	Diabetes (yes VS. no)	Location (head VS. body VS. tail)
miR-103-2	0.528	0.308	0.864	0.137	0.785	0.587	**0.006**	0.28	0.152
miR-125a	0.077	0.143	0.17	0.657	0.108	0.448	0.884	0.83	0.292
miR-126	0.116	0.379	0.845	0.434	0.626	0.313	**0.034**	0.969	0.728
miR-328	0.062	0.626	0.701	0.228	0.309	0.502	0.698	0.833	0.117
miR-340	0.812	0.84	0.925	0.106	0.202	0.156	**0.023**	0.799	0.445
miR-361	**0.016**	**0.03**	0.392	0.976	0.409	**0.01**	0.07	0.311	0.803
miR-374b	0.565	0.57	0.928	0.616	0.869	0.652	**0.022**	0.149	0.389
miR-454	0.596	0.957	0.635	0.614	0.092	0.412	0.07	0.358	0.661
miR-627	0.743	0.842	0.12	0.505	0.356	0.934	**0.005**	0.743	0.128
miR-664	0.507	0.895	0.74	0.085	0.526	0.81	0.085	0.487	0.339
miR-193b	0.91	0.684	0.074	0.321	**0.009**	0.478	0.583	0.44	0.822
miR-21	0.438	0.511	0.56	**0.017**	**0.045**	0.41	0.783	0.814	0.807
miR-584	0.618	0.267	0.886	0.129	0.059	0.899	0.821	0.803	0.577
Risk score	0.573	0.264	0.81	0.932	0.23	0.247	0.112	0.72	0.145

### The miRNA signature risk score as an independent indicator for PC prognosis

**Figure 1:**
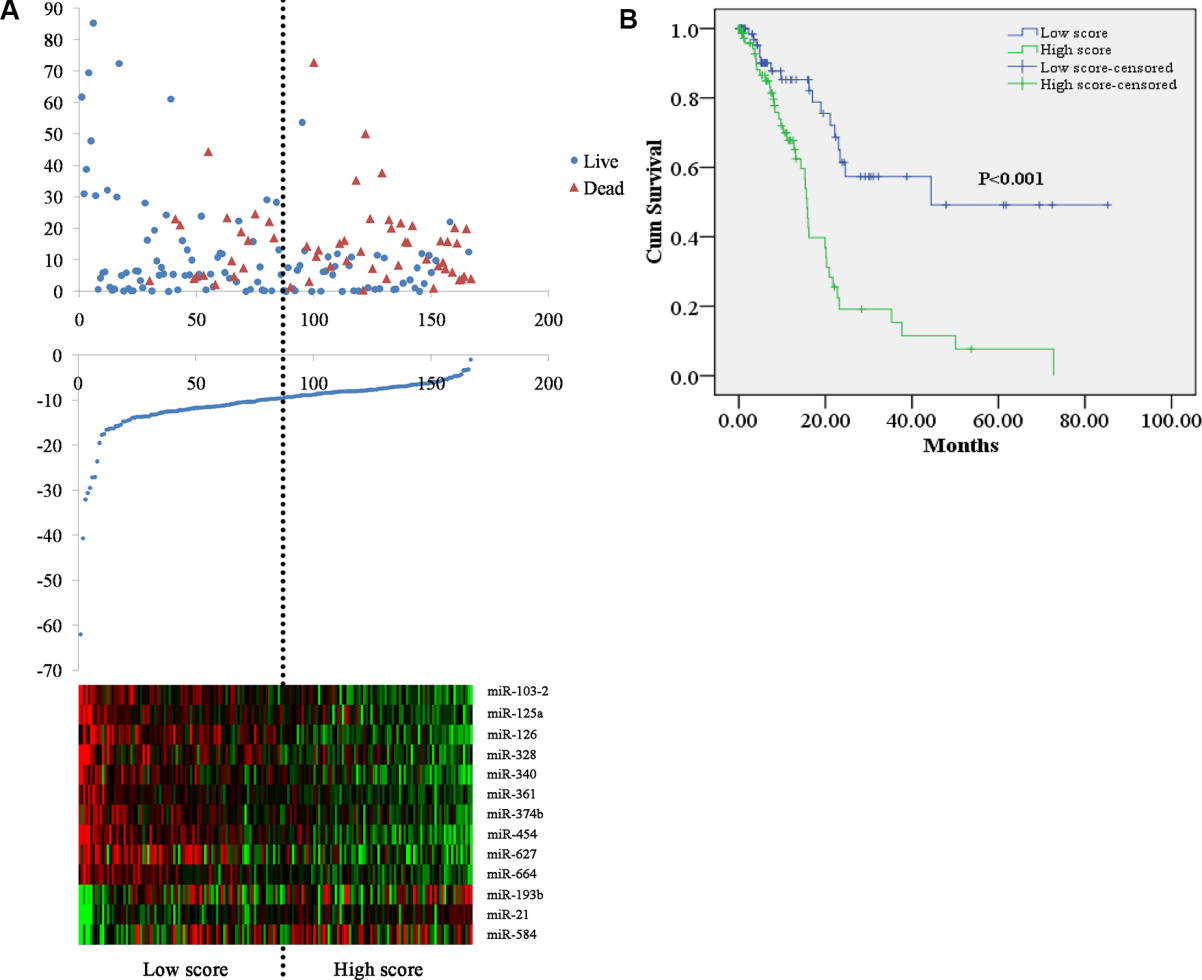
Risk score for miRNA signature and outcome in PC patients (**A**) survival status and duration of cases (Top); risk score of miRNA signature (Middle); low and high score group for the 13 miRNAs (Bottom). (**B**) Kaplan-Meier curve for the low score and high score group.

The risk score for each patient was calculated based on the 13 miRNAs. By applying the median as the cutoff point, 167 PC patients were classified into a high score group (*n* = 83) and low score group (*n* = 84). The heatmap shows that protective miRNAs have high expression in low score group, while the risky miRNAs exhibit high expression in high score group (Figure 1A). And the patients in the high score group suffered significantly worse OS than those in low score group (Figure 1B).

The relationship between clinical features and the miRNA signature risk score was also analyzed (Table 2). However, no significant association of risk score with clinical characteristics was found.

The univariate Cox regression analyses showed that tumor size (*P* = 0.029), tumor grade (*P* = 0.001), residual status (*P* = 0.004), TNM stage (*P* = 0.002), adjuvant radiotherapy (*P* = 0.007), MTT (*P* = 0.004), treatment outcome of the first course (*P* = 0.009) and risk score (*P* < 0.001) were significantly related with OS of PC patients, and the multivariate Cox regression analyses revealed that tumor grade (*P* = 0.009), MTT (*P* = 0.001) and risk score (*P* = 0.017) were independent prognostic factors (Table 3).

**Table 3:** The association of clinical factors and the miRNA signature risk score with OS in PC patients

Variables	Univariate analysis		Multivariate analysis	
	HR (95% CI)	*P* value	HR (95% CI)	*P* value
Gender (female VS. male)	0.799 (0.469, 1.363)	0.411		
Age (≥ 65 VS. < 65)	1.523 (0.899, 2.578)	0.118		
Location (tail VS. body VS. head)	0.586 (0.338, 1.016)	0.057		
Size (≥ 35 VS. < 35; mm)	1.843 (1.063, 3.192)	**0.029**	0.824 (0.427, 1.588)	0.562
Grade (G4 vs. G3 VS. G2 VS. G1)	1.763 (1.247, 2.492)	**0.001**	1.712 (1.141, 2.568)	**0.009**
Residual tumor (yes Vs. no)	2.254 (1.294, 3.927)	**0.004**	1.627 (0.82, 3.225)	0.164
TNM stage (IV VS. III VS. IIB VS. IIA VS. IB VS. IA)	1.723 (1.231, 2.41)	**0.002**	1.459 (0.939,2.267)	0.093
Smoking (smoker VS. non-smoker)	1.13 (0.662, 1.932)	0.654		
Drinking (drinker VS. none-drinker)	0.76 (0.358, 1.614)	0.476		
Diabetes (yes VS. no)	0.909 (0.467, 1.767)	0.778		
Adjuvant radiotherapy (yes VS. no)	0.337 (0.152, 0.746)	**0.007**	0.653 (0.24, 1.773)	0.403
MTT (yes VS. no)	0.453 (0.265, 0.774)	**0.004**	0.256 (0.117, 0.562)	**0.001**
Treatment outcome (SD + PD VS. CR + PR)	2.25 (1.222, 4.145)	**0.009**	1.427 (0.733, 2.779)	0.296
Risk score (high VS. low)	3.196 (1.795, 5.689)	**< 0.001**	2.641 (1.194, 5.845)	**0.017**

HR: hazard ratio; CI: confidence interval; SD: stable disease; PD: progressive disease; CR: complete response; PR: partial response.

### The miRNA signature risk score as a predicator for treatment outcome

**Figure 2:**
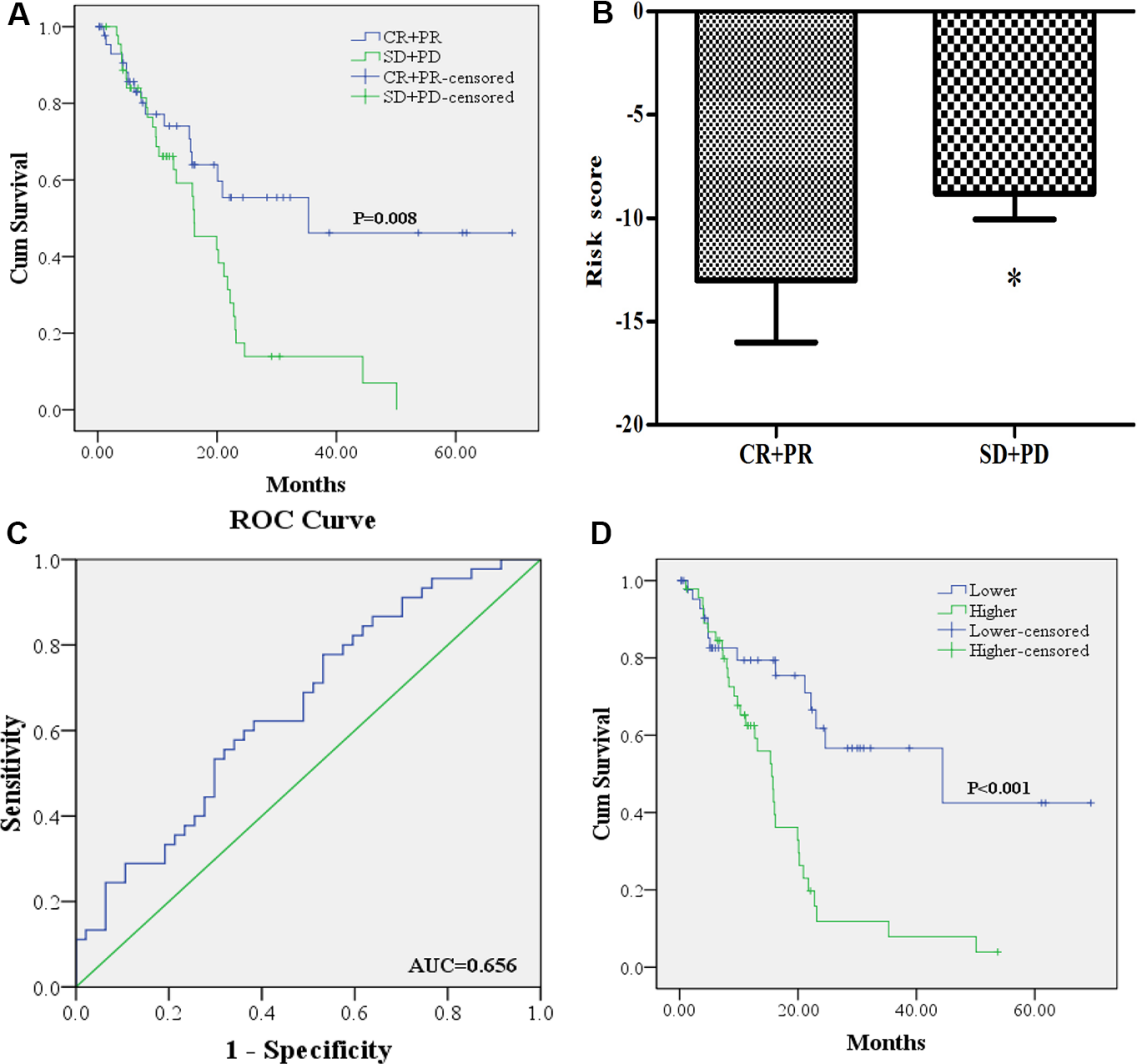
The association of the miRNA signature risk score with treatment outcome (**A**): 47 PC patients who achieved CR or PR (CR + PR) after treatment of first course had better OS than the 45 patients with SD or PD (SD + PD). (**B**) risk score of PC patients in CR + PR group was lower than those in SD + PD group. (**C**) receiver-operating characteristic (ROC) curve analysis of the risk score to discriminate patients with CR + PR from those with SD + PD. (**D**) 46 PC patients with risk score higher than the cutoff value had worse OS than the 46 cases lower than the score.

Better OS could be found in the 47 PC patients that achieved CR or PR after treatment of first course than the 45 patients with SD or PD (Figure 2A). We compared the difference of risk score in the two groups (CR + PR VS. SD + PD) and applied the receiver operating characteristic (ROC) curve to evaluate whether the risk score could predict treatment outcome of first course. As shown in Figure 2B, risk score of PC patients in CR + PR group was significantly lower than that in SD + PD group. The area under the curve (AUC) was 0.656 (95% confidence interval (CI): 0.546–0.767). The sensitivity and specificity of risk score for predicting treatment outcome was 0.622 and 0.617 when optimal cutoff value of −9.27 was used. And the 46 PC patients with risk score higher than the cutoff value suffered worse OS than the 46 cases lower than the score (Figure 2D).

**Figure 3:**
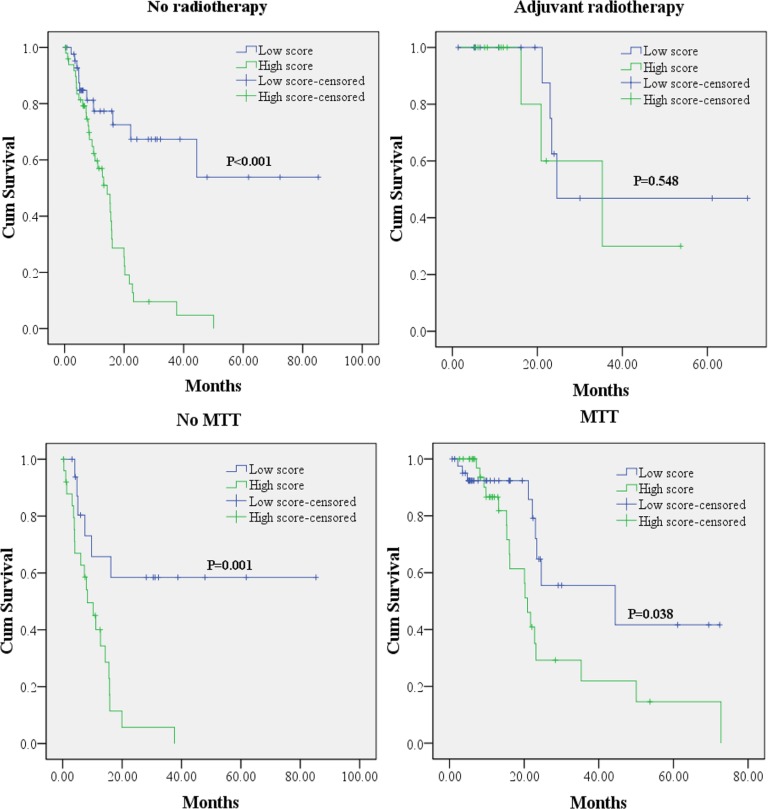
Kaplan-Meier survival curves for PC patients with high and low risk score in subgroup analyses according to different treatment modalities MTT: molecular targeted therapy.

In addition, we also explored the prognostic value of the miRNA signature in the subgroups by different treatment procedures. Kaplan-Meier curves (Figure 3) showed that lower risk score could predict a better OS in patients without but not cases with adjuvant radiotherapy. As patients received MTT or not, lower risk score could act as a favorable indicator in the prognosis of PC patients.

## DISCUSSION

PC is one of the deadliest of the solid malignancies [1]. Besides the highly aggressive properties of PC, the lack of specific biomarkers for the diagnosis, monitoring therapy and prognosis might also be responsible for the low survival rate.

By using prognostic biomarkers, PC patients could be defined as different tumor subgroups and obtained individualized anti-cancer therapy which might lead to improved survival [19]. In the present study, we identified a miRNA signature which could independently predict OS in PC patients using the data retrieved from TCGA. To obtain miRNAs significantly correlated with OS, the univariate Cox proportional hazards regression with significance level set as 0.001 was performed on 339 miRNAs from 1046 miRNAs according to the criterion. A total of 13 miRNAs including 10 protective miRNAsand 3 risky miRNAs were identified. The relationship between the 13 miRNAs and clinical features revealed that miR-21 and miR-193b might be more closely associated with PC. However, a single miRNA is less sensitive and specific than a panel of miRNAs [18, 20]. Thus, we developed a risk score by combination of the 13 miRNAs and found that the risk score could independently predict OS in PC patients. In addition, the risk score could also act as an indicator for treatment outcome of first course with sensitivity of 0.622 and specificity of 0.617. In our study, MTT was an independently favorable factor for PC patients. PC patients could also benefit from adjuvant radiotherapy. We conducted subgroup analyzes to assess whether the risk score could predict OS in cases with different treatment modalities. And the results showed that except in the patient who received radiotherapy, the higher risk score also indicated worse OS in PC patients without radiotherapy and those whether received MTT. Thus, we considered that PC patients with higher score should take treatment of radiotherapy and MTT, and those who received MTT and were identified in the high score group might benefit from radiotherapy.

A number of miRNAs have been reportedly correlated with survival of PC. As the well known onco-miRNA in various cancers [21], miR-21 was studied most times as a prognostic marker in PC patients [14, 19]. In our study, we demonstrated that high miR-21 was significantly related with worse OS. This result was consistent with previous studies. In addition, the expression level of miR-21 could also reflect the difference of tumor size and stage. Recently, Lee et al. [22] identified a prognostic miRNA signature including 7 miRNAs from 221 formalin-fixed paraffin-embedded (FFPE) surgical specimens using nCounter microRNA expression assays. Among the 7 miRNAs, miR-99a, miR-150, miR-194, miR-375 and miR-664 were related with favorable OS, while miR-342-3p and miR-487b may indicate worse OS in PC patients. Our study also yielded the similar result that higher expression of miR-664 was significantly associated with better OS. It was reported that miR-126 was down-regulated in PC tissues and could act as a tumor suppressor in PC by inhibiting ADAM9, KRAS and CRK [23–25] which was in accordance with our findings.

Tian et al. [26] recently reported that subjects with low serum miR-103 were observed to have a higher risk for PC. While the study of Piepoli et al. showed that miR-103 was up-regulated in PC tissues and was one of driving miRNAs in PC [27]. Interestingly, Yang and colleagues analyzed differentially expressed miRNAs from 3 expression profiling studies including 60 PC and 21 normal tissue samples and found miR-125a was one of up-regulated miRNAs in PC. MiR-193b was found to be decreased in PC and could inhibit proliferative, migratory, and invasive ability of PC cells [28, 29]. In another study by Thorns et al., miR-193b was identified to be up-regulated in both tissue and serum samples and might be a potential biomarker in pancreatic neuroendocrine neoplasms. These findings were not consistent with our results that miR-103 and miR-125a were identified as protective miRNAs and miR-193b as a risky miRNA which was also positively related with tumor stage. We assumed that these miRNAs might also be involved in other complex mechanisms such as drug metabolism and treatment resistance and then affect OS in PC patients enrolled in our study. The exact mechanisms are warranted to be investigated in the future. The other 7 miRNAs were not explored so widely in PC and further researches are needed to explore their complex molecular mechanisms.

Compared with previous studies, our study used data from TCGA with high throughput analysis of miRNAs. Up to 1046 miRNAs were initially included in the present study which could provide a more comprehensive scan. Furthermore, the significance level was set as 0.001 to control the false discovery rate. By combination of the 13 identified miRNAs, the miRNA signature risk score could act as an independent predictor in PC. The performance of our generated prognostic model was also validated in leave-one-out cross validation (LOOCV) model [22, 30, 31] (Supplementary Figure S1 online). No significant association of the miRNA signature risk score with clinical features such as TNM stage might provide another view on the prognosis of PC patients independent of the existing assessment system. It might be helpful in the improvement of clinical outcome through further classification of PC patients.

However, some limitations should be considered. First, the number of PC patients enrolled in our study was 167. The mean time of follow-up was 12.8 months. The study that included more participants with longer follow-up time is warranted to validate our findings in the future. Second, pancreas adenocarcinoma covers the most in PC. 161 of 167 patients enrolled in our study were classified as pancreas adenocarcinoma. When excluded the data of the other 6 patients diagnosed as pancreas colloid carcinoma or pancreas undifferentiated carcinoma, the results did not change. However, the prognostic value of the miRNA signature in more subtypes of PC is warranted to be evaluated in the future. Third, some miRNAs identified in our study showed inconsistent roles when compared with previous studies. The complicated effects and mechanisms of these miRNAs are needed to be further studied.

In conclusion, by analyzing the genome-wide miRNA expression profiles from TCGA, we identified a panel of miRNA signature including 13 miRNAs, which could act as an indicator for treatment outcome and could be served as an independent factor in prognosis of PC.

## MATERIALS AND METHODS

### Expression profiles and sample information

The miRNA expression microarray data (Level 3) and clinical data for PC patients (up to March 17, 2015) were downloaded from TCGA data portal. The expression of 1046 miRNAs in PC samples was analyzed on IlluminaHiSeq miRNASeq platform. The subjects without miRNA sequence data or clinical data and those had history of other malignancies were excluded. Thus, a total of 167 PC subjects with the corresponding clinical data including gender, age, tumor location, tumor size, grade, residual status, AJCC TNM stage, smoking status, drinking status, diabetes status, adjuvant radiotherapy, molecular targeted therapy (MTT) status and treatment outcome of first course were enrolled in our study. The end-point in our study was OS. As the data were retrieved from TCGA, further approval by an ethics committee was not required. Data procession was conducted according to the TCGA human subject protection and data access policies.

### Statistical analysis

The expression level of 1046 miRNAs was presented as reads per million (RPM) miRNA mapped data. The miRNAs which were less than 1 RPM in exceeded 10% of all subjects were eliminated using BRB array tools package (version 4.4.0; National Cancer Institute, Bethesda, MD, USA) that was developed by Richard Simon and the BRB-ArrayTools Development Team [32]. And the expression level of each miRNA was log2 transformed for further analysis. The univariate Cox proportional hazards regression with significance level set as 0.001 was performed to find out the miRNAs significantly associated with OS. A total of 13 miRNAs were identified and were divided into risky (with a hazard ratio (HR) for death greater than 1) and protective (based on a HR for death less than 1) types. A risk score formula for predicting OS was developed based on a linear combination of the expression level multiplied regression coefficient derived from the univariate cox regression model (β) [33, 34]: risk score = expgene1*βgene1 + expgene2*βgene2 + … expgenen*βgenen. By utilizing the median risk score as the cutoff point, PC patients were divided into high score and low score groups.

Univariate Cox proportional hazards regression analyses were performed to explore the effects of clinical features and the risk score on OS of PC patients. Each predictor identified via univariate analysis was further assessed by multivariate Cox proportional hazards regression analysis. Survival curves were estimated by the Kaplan-Meier and log-rank method.

The relationship between the miRNA signature and clinical features were assessed by Chi-square test. ROC curve was used to evaluate the predictive value of the risk score for patients' outcome after first course of treatment. Statistical significance was defined as a two-sided *P value* < 0.05 unless specifically indicated. The statistical analyses were performed with the use of BRB-Array Tools and SPSS16.0 software (SPSS Inc., Chicago, IL, USA), as appropriate.

## SUPPLEMENTARY MATERIALS



## Abbreviations

miRNA: microRNA
PC: pancreatic cancer
TCGA: The Cancer Genome Atlas
OS: overall survival
CA 19-9: carbohydrate antigen 19–9
MTT: molecular targeted therapy
RPM: reads per million
HR: hazard ratio
ROC: receiver operating characteristic
SDV: standard deviation
CR: complete response
PR: partial response
SD: stable disease
PD: progressive disease
AUC: area under the curve
CI: confidence interval
FFPE: formalin-fixed paraffin-embedded
LOOCV: leave-one-out cross validation
