# Supplementary material for: A panel of 13-miRNA signature as a potential biomarker for predicting survival in pancreatic cancer
Source: Oncotarget. 2016 Sep 8;7(43):69616–24. doi: 10.18632/oncotarget.11903 (PMC5342502; doi:10.18632/oncotarget.11903)
Supplement: Supplementary file 1 [file oncotarget-07-69616-s001.pdf]

A panel of 13-miRNA signature as a potential biomarker for predicting survival in pancreatic cancer

Supplementary Materials

Supplementary Table S1: 13 miRNAs significantly related with OS in PC patients

miRNA	HR (95% CI)	*P* value
miR-103-2	0.802 (0.705, 0.913)	8.34E-04
miR-125a	0.998 (0.997, 0.999)	1.58E-04
miR-126	0.999 (0.999, 1)	6.94E-04
miR-328	0.956 (0.932, 0.981)	7.43E-04
miR-340	0.931 (0.894, 0.97)	6.41E-04
miR-361	0.995 (0.993, 0.998)	4.41E-04
miR-374b	0.982 (0.973, 0.992)	4.30E-04
miR-454	0.824 (0.736, 0.922)	7.26E-04
miR-627	0.46 (0.3, 0.707)	3.94E-04
miR-664	0.959 (0.936, 0.982)	5.91E-04
miR-193b	1.01 (1.005, 1.015)	1.97E-04
miR-21	1 (1, 1)	7.50E-05
miR-584	1.006 (1.003, 1.01)	7.87E-04

HR: hazard ratio; CI: confidence interval.

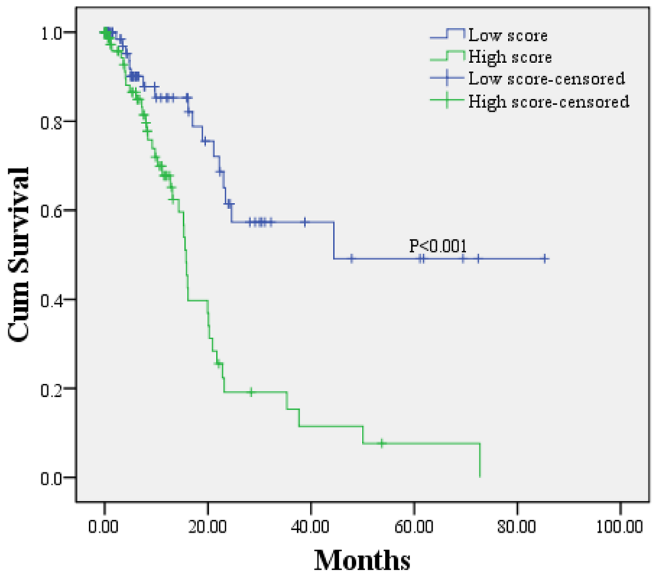

Supplementary Figure S1: Kaplan-Meier curve for high and low score groups classified by the miRNA signature from LOOCV procedure.
